# Physician Experiences and Prescribing Preferences for Targeted B-complex and Vitamin C Supplementation in Daily Clinical Practice: A National KAP Survey in India

**DOI:** 10.7759/cureus.115073

**Published:** 2026-08-24

**Authors:** Vikas Oswal, Vinay Purohit, Poonam Shah

**Affiliations:** 1 Pulmonology, Vikas Nursing Home, Mumbai, IND; 2 Medical Affairs, Consumer Health Division, Bayer Pharmaceuticals Private Limited, Thane, IND

**Keywords:** aphthous ulcers, b-complex vitamins, immune function, knowledge attitude practice survey, micronutrient supplementation, neuropathy, prescribing patterns, vitamin c, wound healing

## Abstract

Background

Water-soluble vitamins, particularly B-complex vitamins and vitamin C, play essential roles in energy metabolism, immune function, and tissue repair. Despite widespread clinical use, variability in evidence across indications and prescribing practices necessitates evaluation of physician perspectives. This study aimed to assess knowledge, attitudes, and practices (KAP) of clinicians regarding Becozym C Forte^®^ in routine clinical settings.

Methods

A cross-sectional KAP survey was conducted among 212 physicians to evaluate perceptions, prescribing behaviors, and clinical experiences related to targeted B-complex plus vitamin C formulations. The survey included domains assessing clinical efficacy, product preference across multiple scenarios, and comparative perceptions versus multivitamin-multimineral formulations. Responses were analyzed descriptively using proportions and frequency distributions.

Results

A high level of agreement was observed regarding improved tolerance and adherence with targeted formulations (209, 98.58%). Clinicians reported perceived benefits in fatigue, with over 90% indicating improvement in at least half of patients. High effectiveness was also reported for mouth ulcers (156, 73.58% reporting >75% improvement), while more than 80% of clinicians perceived improvement in neuropathy-related symptoms in at least 50% of patients. Focused vitamin supplementation was preferred over multivitamin-multimineral products in suspected water-soluble vitamin deficiencies (185, 87.26%) and across multiple clinical scenarios, including recovery, stress-related fatigue, and antibiotic use. Improved energy levels (183, 86.32%) and better tolerability were the most commonly perceived advantages.

Conclusion

Physicians demonstrated a strong preference for targeted B-complex and vitamin C supplementation across diverse clinical conditions, supported by perceived benefits in energy, tolerability, and symptom relief. While these perceptions align with known biological roles of these micronutrients, variability in supporting clinical evidence highlights the need for cautious interpretation and further well-designed studies to strengthen evidence-based use in routine practice.

## Introduction

Micronutrients are essential for maintaining normal physiological and metabolic functions, with water-soluble vitamins - including the B-complex group and vitamin C - playing a critical role in multiple cellular processes. Humans require adequate intake of these vitamins from dietary sources, as they are not synthesized endogenously, underscoring their importance in maintaining physiological homeostasis [1,2]. B-complex vitamins function as coenzymes in a wide range of biochemical pathways, including energy production, DNA and RNA synthesis, methylation reactions, and neurotransmitter synthesis, thereby supporting optimal neurological and systemic function [1]. These vitamins exhibit closely interrelated biological roles, suggesting that a balanced supply of multiple water-soluble micronutrients may be important for optimal cellular functioning.

The importance of B-complex vitamins extends across all stages of life, from early development to aging, influencing processes such as growth, neurological function, and metabolic regulation. Deficiencies in these vitamins have been associated with a spectrum of clinical manifestations, including anemia, neurological dysfunction, and impaired cognitive performance, highlighting their essential role in human health [3]. In parallel, vitamin C is an essential micronutrient with pleiotropic biological functions, including roles as an antioxidant and as a cofactor for biosynthetic and gene regulatory enzymes [2]. It contributes to immune defense by supporting epithelial barrier integrity and enhancing the function of immune cells, including phagocytes and lymphocytes, thereby playing a critical role in host defense mechanisms [2].

In clinical practice, vitamin supplementation - particularly B-complex formulations, often in combination with vitamin C - constitutes a substantial proportion of prescriptions. Evidence indicates that vitamins account for approximately 24%-25% of prescribed medications in certain settings, frequently being used for a variety of clinical and non-specific indications [4].

From a mechanistic perspective, B vitamins are integral to cellular energy metabolism, acting as essential cofactors in mitochondrial processes responsible for adenosine triphosphate (ATP) production [1]. Deficiencies in these vitamins may impair metabolic efficiency and contribute to fatigue-related symptoms. Clinical evidence suggests that B-complex supplementation may improve exercise-related fatigue and metabolic performance in certain populations [5]. Complementing these effects, vitamin C contributes to cellular redox balance, protects biomolecules from oxidative damage, and supports metabolic processes such as carnitine synthesis, which is important for energy production [6]. Together, these roles provide a strong biological rationale for the combined use of B-complex vitamins and vitamin C in conditions characterized by fatigue, stress, and recovery.

In addition to their metabolic roles, B vitamins have been extensively studied in relation to neurological health, particularly peripheral neuropathy. Evidence indicates that deficiencies in vitamins such as B12 are associated with neuropathic symptoms, potentially mediated through mechanisms involving impaired methylation, accumulation of homocysteine, and disruption of myelin integrity [6]. While some studies suggest that B-vitamin supplementation may contribute to symptom improvement, the overall evidence remains heterogeneous, underscoring the need for careful clinical interpretation.

Given the widespread use of micronutrient supplementation in clinical practice, particularly combinations of B-complex vitamins with vitamin C, and the variability in supporting evidence across different clinical indications, there is a need to better understand physician perspectives, prescribing behaviors, and real-world clinical preferences. Knowledge-attitude-practice (KAP) surveys provide valuable insights into how clinicians interpret available evidence and translate it into therapeutic decision-making.

In this context, this study aimed to systematically assess Indian physicians’ knowledge, attitudes, and prescribing practices regarding targeted B-complex and vitamin C supplementation (Becozym C Forte®), focusing on perceived clinical efficacy, product preferences across common clinical scenarios, and comparative perceptions versus multivitamin-multimineral formulations.

## Materials and methods

This study was designed as a cross-sectional, questionnaire-based KAP survey conducted among practicing physicians in India.

A structured questionnaire was developed to assess physicians’ perspectives on the use, efficacy, safety, and clinical preferences related to B-complex and vitamin C supplementation compared with multivitamin-multimineral formulations across various clinical scenarios (see Appendix 1).

The survey was administered electronically using a secure, web-based platform. Invitations containing a unique survey link were distributed to eligible physicians via email. To ensure data integrity and prevent duplicate responses, access to the survey was restricted through one-time password (OTP)-based authentication. The survey was built and hosted on a dedicated, custom-developed platform (developed by a third-party digital agency, Insignia Communications, on their domain); access was link- and OTP-restricted to invited physicians only. Physicians who had not completed the survey were followed up periodically until completion or closure of the survey period.

A total of 212 physicians were invited to participate in the survey; all 212 (100%) completed the survey with fully valid responses. Prior to participation, all respondents provided informed digital consent. Participation was voluntary, and responses were anonymized to maintain confidentiality.

The study protocol, including the survey questionnaire and methodology, was reviewed and approved by the Samarth Independent Ethics Committee (approval date: January 25, 2026) prior to the initiation of the study. The survey was conducted from 29th January 2026 to 24th February 2026 in compliance with the principles of the Declaration of Helsinki. The sponsor had no role in data collection, data analysis, or interpretation of survey responses.

Statistical analysis

Descriptive statistical analysis was performed, and results were summarized as frequencies and percentages. No inferential statistical testing was performed because the survey was exploratory and descriptive in nature.

## Results

A total of 212 physicians participated in the survey. The dataset reflects physician-reported experiences across multiple domains, including perceived efficacy, patient outcomes, and prescribing preferences in varied clinical contexts such as fatigue, neuropathy, recovery, and micronutrient deficiencies, thereby enabling a holistic evaluation of current practice patterns (Table 1).

**Table 1:** Respondent characteristics

Speciality	N (%)
General Practitioner	108 (50.9)
Consulting Physician	81 (38.2)
MS	10 (4.7)
Other	8 (3.8)
Gynecologist	5 (2.4)
Total	212

Among the 212 respondents, 70% were based in private clinic settings and 30% in hospital or institutional setups. The cohort represented a broad spectrum of clinical experience: 52 respondents (24.5%) had 5-10 years of experience, 65 (30.7%) had 11-20 years, and the largest group, 95 respondents (44.8%), had more than 20 years of clinical experience - indicating a predominantly senior, well-experienced physician cohort.

Clinical efficacy and patient outcomes

Tolerance and Adherence

**Figure 1:**
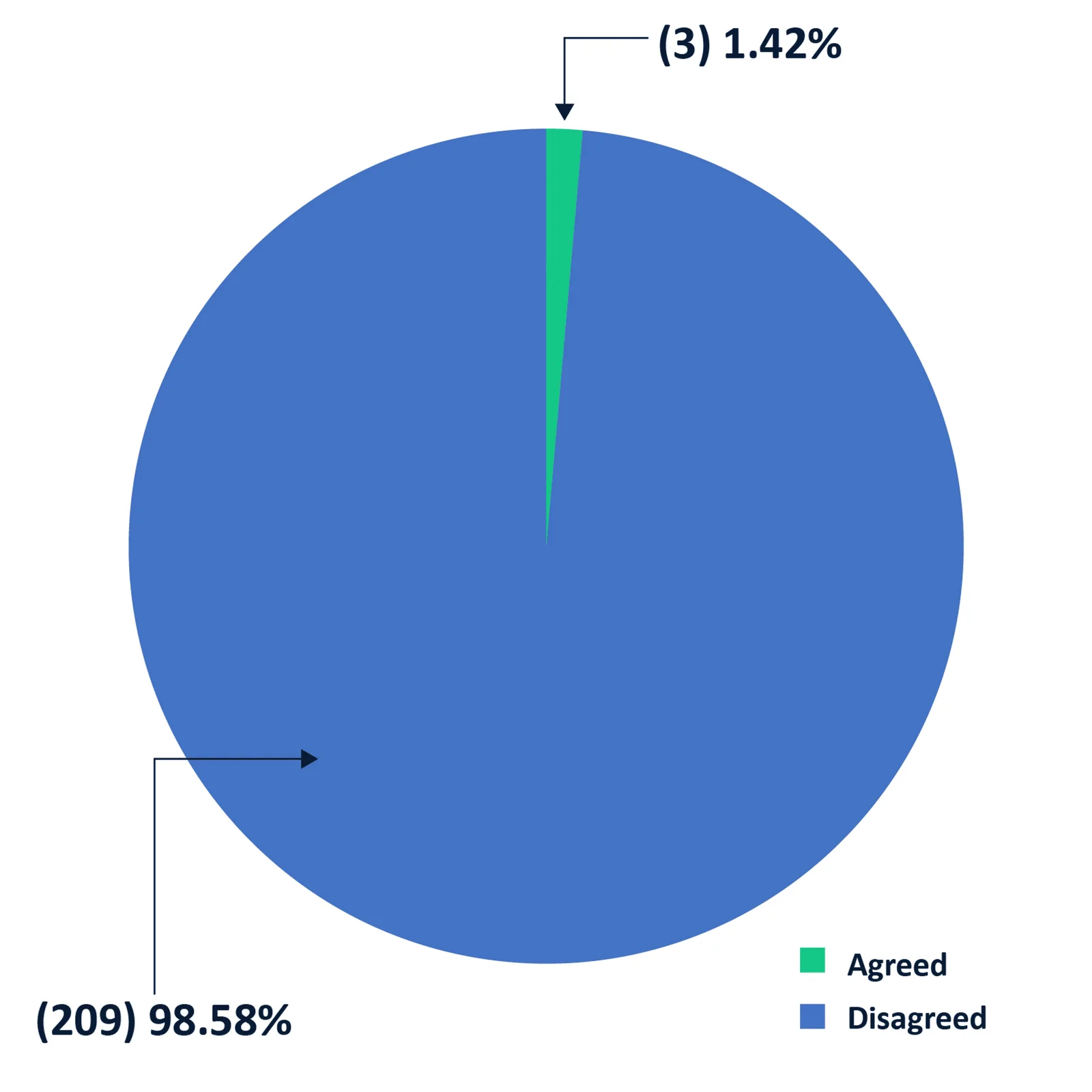
Clinician agreement on improved tolerance and adherence with focused B-complex + vitamin C formulations

A high proportion of clinicians (98.58%, n=209) agreed that a focused B-complex plus vitamin C formulation may offer improved patient tolerance and adherence compared with multivitamin-multimineral products, while 1.42% (n=3) disagreed. This finding indicates a strong physician-perceived benefit of targeted formulations, although it reflects subjective clinical experience rather than direct comparative outcome data (Figure 1).

Fatigue Relief

Clinicians observed substantial improvement in fatigue symptoms within two to three weeks of B-complex therapy. Specifically, 45.28% (n=96) indicated that more than 75% of patients experienced relief, while an equal proportion (45.28%, n=96) reported improvement in 50%-75% of patients. Only 8.02% (n=17) and 1.42% (n=3) reported improvement in 25%-50% and <25% of patients, respectively. Overall, more than 90% of clinicians perceived improvement in fatigue symptoms in at least half of their patients, indicating a strong perceived benefit in this population (Table 2).

**Table 2:** Distribution of clinician responses on fatigue relief rates within 2-3 weeks of B-complex therapy

Response Category	N (%)
>75% patients improved	96 (45.28)
50-75% patients improved	96 (45.28)
25-50% patients improved	17 (8.02)
<25% patients improved	3 (1.42)
Total	212

Mouth Ulcer Relief

B-complex supplementation was widely perceived as effective for the management of mouth ulcers within seven days. A majority of clinicians (73.58%, n=156) perceived it as highly effective (>75% of patients responding), while 24.06% (n=51) considered it moderately effective (50%-75% response). Only 2.36% (n=5) reported limited effectiveness (25%-50%), and none reported ineffectiveness. These findings suggest a consistently favorable clinical perception of B-complex supplementation in oral mucosal conditions (Table 3).

**Table 3:** Clinician effectiveness ratings for B-complex in mouth ulcer relief within 7 days

Effectiveness	N (%)
Highly effective (>75%)	156 (73.58)
Moderately effective (50-75%)	51 (24.06)
Somewhat effective (25-50%)	5 (2.36)
Not effective (<25%)	0 (0)
Total	212

Neuropathy and Nerve-Related Symptoms

Clinicians reported meaningful improvement in neuropathy or nerve-related symptoms with B-complex supplementation. Approximately 34.91% (n=74) indicated that more than 75% of patients experienced improvement, while 47.64% (n=101) reported improvement in 50%-75% of patients. Smaller proportions reported improvement in 25%-50% (14.62%, n=31) and <25% (2.83%, n=6) of patients. Overall, more than 80% of clinicians observed improvement in at least half of their patients, reflecting a strong perceived role of B-complex supplementation in nerve-related conditions (Table 4).

**Table 4:** Neuropathy improvement

Response Category	N (%)
>75% patients improved	74 (34.91)
50-75% patients improved	101 (47.64)
25-50% patients improved	31 (14.62)
<25% patients improved	6 (2.73)
Total	212

Indications for Targeted B-complex Plus Vitamin C

Clinicians reported a broad range of clinical indications for prescribing B-complex and vitamin C combination without additional multiminerals. The most frequently cited indication was mouth ulcers (84.91%, n=180), followed by general fatigue (77.36%, n=164). Acute illness recovery and immune support were each reported by 69.81% (n=148) of clinicians, while nerve health was cited by 64.15% (n=136). Additional indications included wound healing (51.42%, n=109), cognitive health (49.06%, n=104), anemia (33.49%, n=71), and stress management (32.08%, n=68). These findings indicate that clinicians preferentially utilize targeted formulations across both symptomatic and recovery-related clinical contexts (Table 5).

**Table 5:** Indications for targeted B-complex plus vitamin C Multiple responses, n=212

Condition	N (%)
Mouth ulcers	180 (84.91)
General fatigue	164 (77.36)
Immune support	148 (69.81)
Acute illness recovery	148 (69.81)
Nerve health	136 (64.15)
Wound healing	109 (51.42)
Cognitive health	104 (49.06)
Anemia	71 (33.49)
Stress management	68 (32.08)

Formulation Preferences

When prescribing for suspected deficiencies of water-soluble vitamins, a substantial majority of clinicians (87.26%, n=185) preferred focused micronutrient formulation, whereas only 12.74% (n=27) favored multivitamin-multimineral complexes. This pattern suggests a strong inclination toward focused supplementation when deficiency profiles are clinically suspected (Table 6).

**Table 6:** Preference in water-soluble vitamin deficiencies

Option	N (%)
Focused B-complex + Vitamin C	185 (87.26)
Multivitamin-multimineral	27 (12.74)
Total	212

Scenario-based prescribing preferences

Post-surgical or Post-injury Recovery

**Figure 2:**
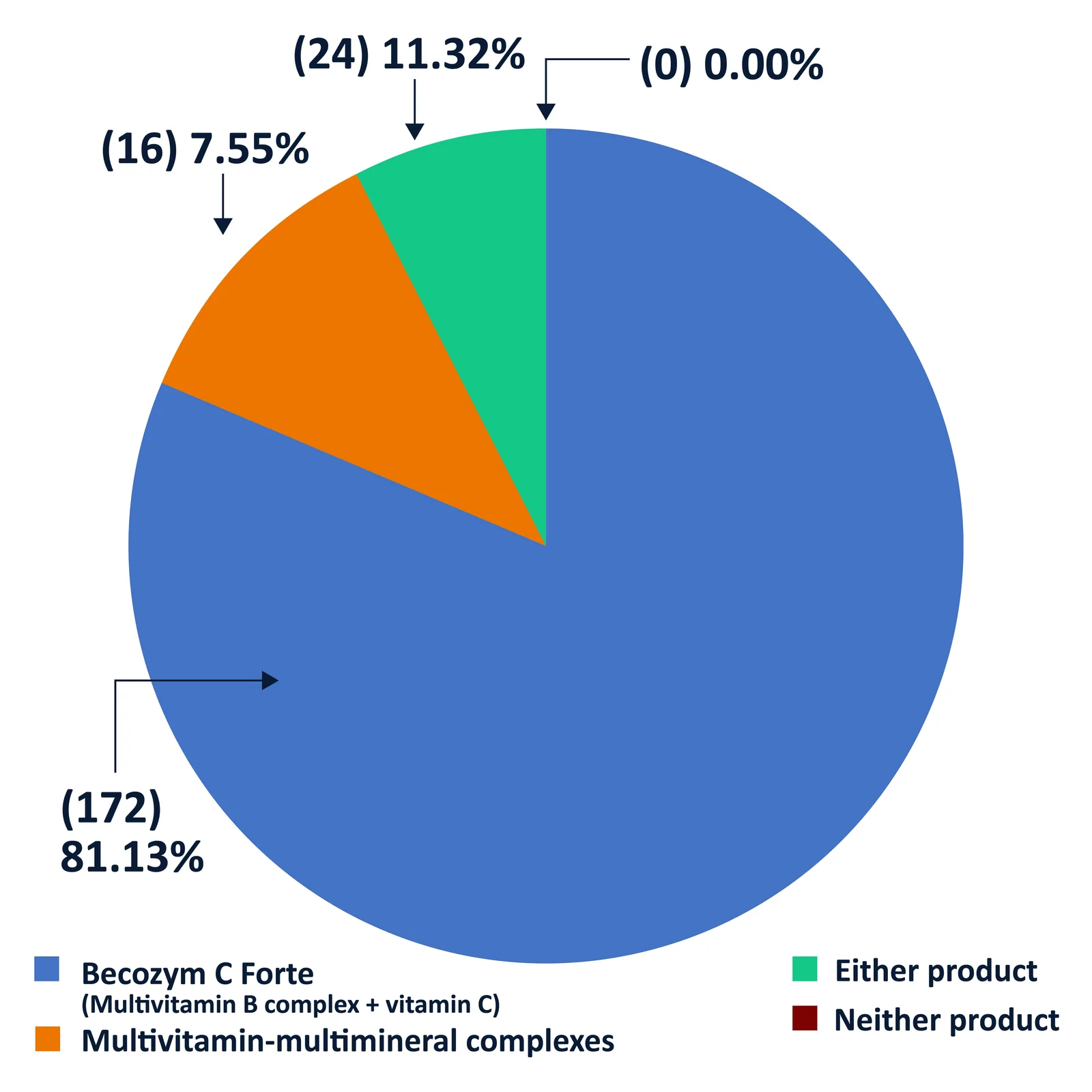
Prescribing preference for post-surgical/post-injury wound healing support

For patients requiring wound healing support, 81.13% (n=172) of clinicians preferred combined multivitamin preparations, compared with 7.55% (n=16) who preferred multivitamin-multimineral products; 11.32% (n=24) reported no specific preference (Figure 2).

Stress-Related Fatigue and Cognitive Concerns

**Figure 3:**
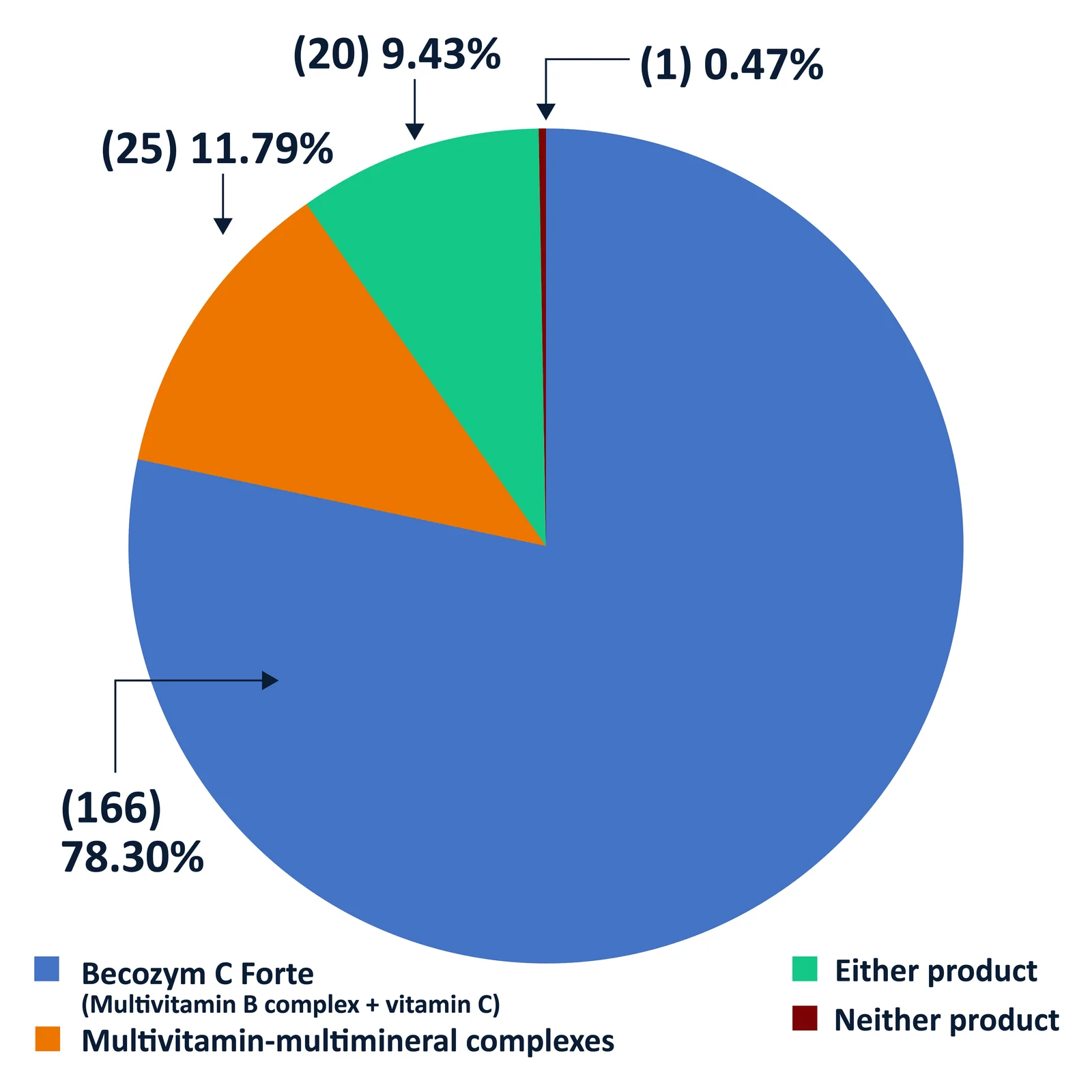
Prescribing preference for stressed professionals with fatigue and concentration issues

In stressed individuals presenting with fatigue and concentration difficulties, 78.30% (n=166) of clinicians preferred targeted formulations, while 11.79% (n=25) favored multivitamin-multimineral products and 9.43% (n=20) reported no preference. Only 0.47% (n=1) indicated neither option (Figure 3).

Elderly Patients With Poor Dietary Intake

**Figure 4:**
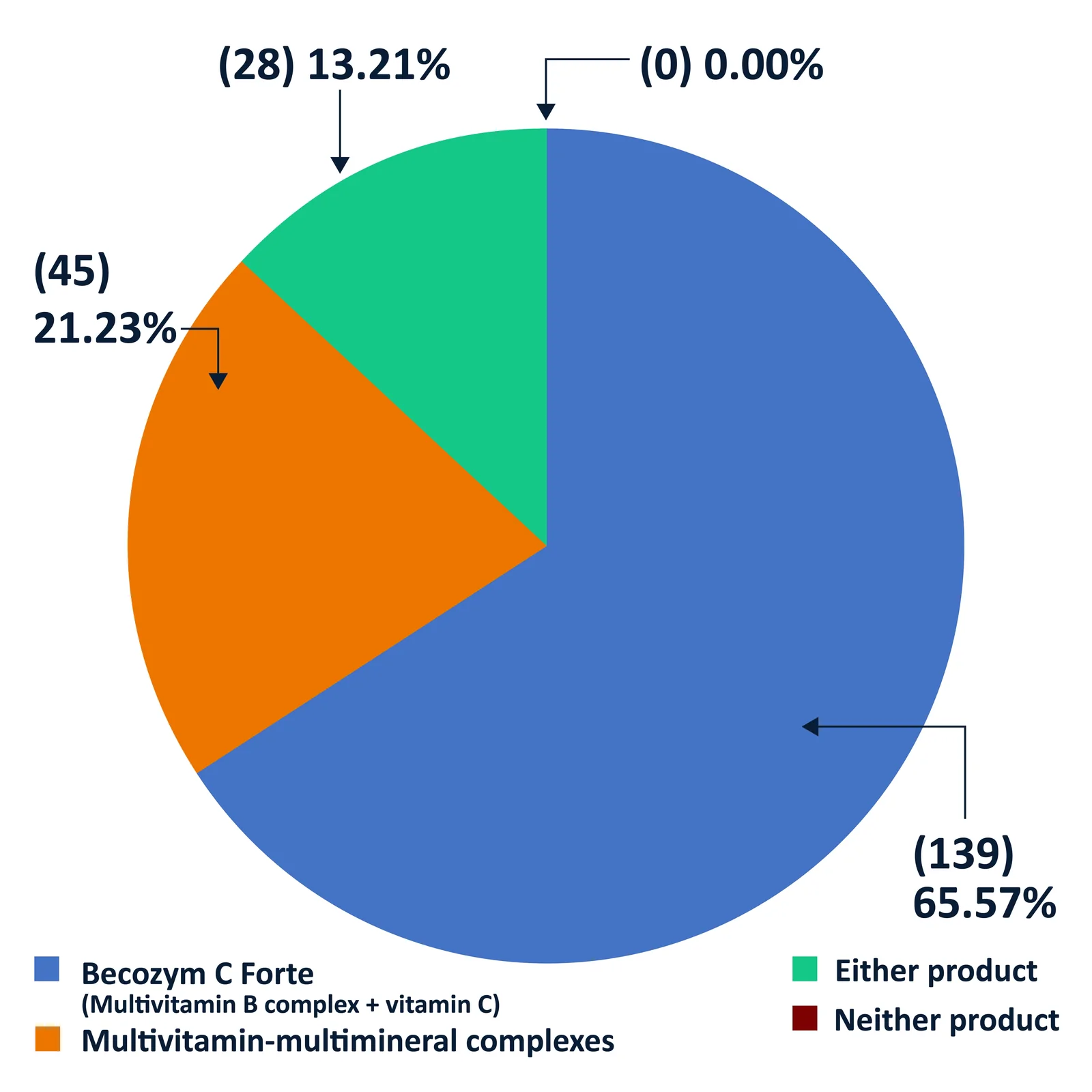
Prescribing preference for elderly patients with poor dietary intake

Among elderly patients with suboptimal nutritional intake, 65.57% (n=139) of clinicians preferred targeted B-complex plus vitamin C formulations. A relatively higher proportion compared with other scenarios (21.23%, n=45) favored multivitamin-multimineral products, while 13.21% (n=28) expressed no preference (Figure 4).

Patients on Long-Term Medications

**Figure 5:**
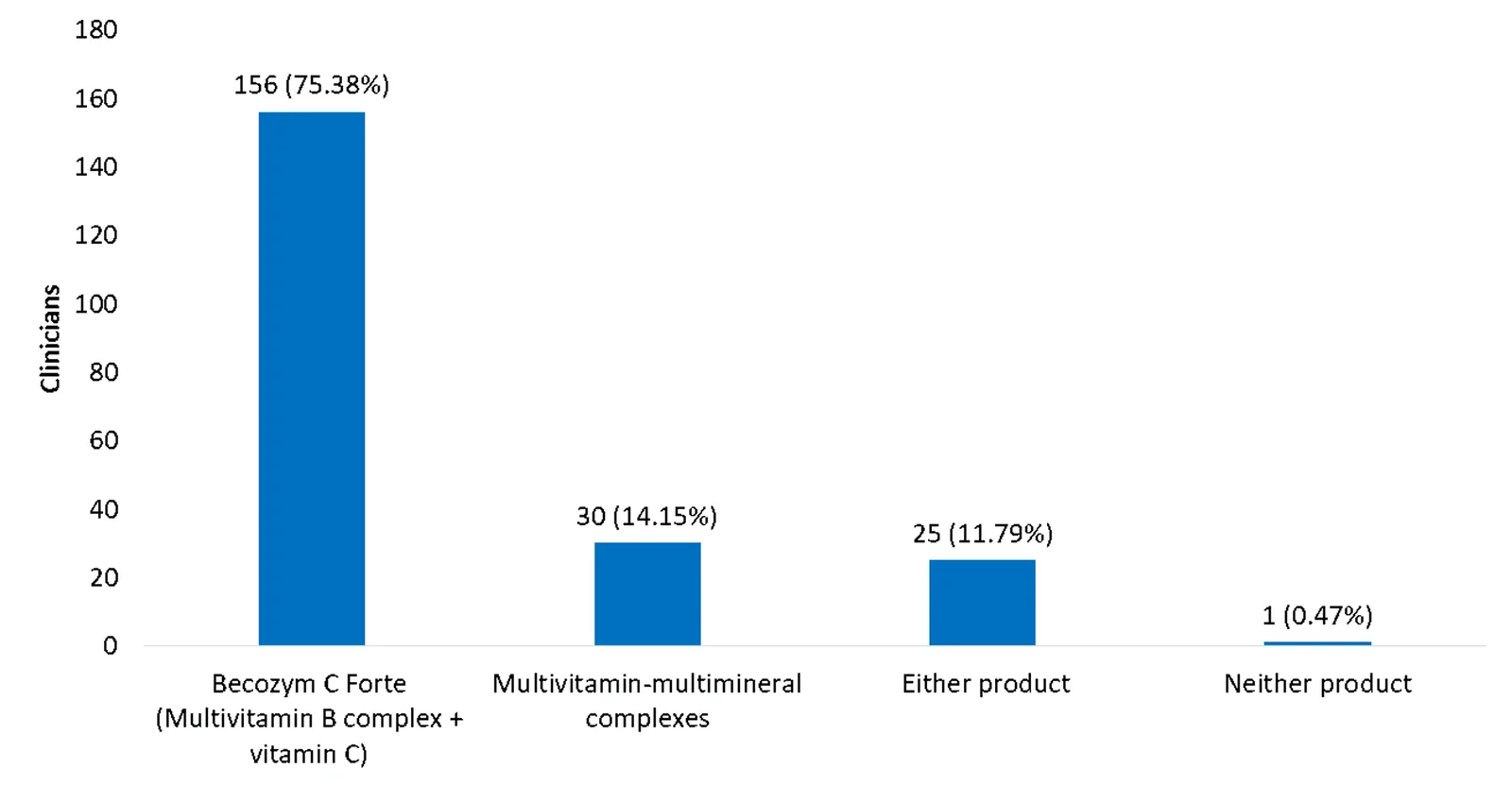
Prescribing preference for patients on long-term medications (metformin, PPIs, and anticonvulsants) PPIs: Proton Pump Inhibitors

For patients receiving long-term medications associated with nutrient depletion (e.g., metformin, proton pump inhibitors, and anticonvulsants), 73.58% (n=156) of clinicians preferred targeted formulations, compared with 14.15% (n=30) for multivitamin-multimineral products and 11.79% (n=25) reporting no preference (Figure 5).

Convalescing Patients During Acute Illness or Infection

**Figure 6:**
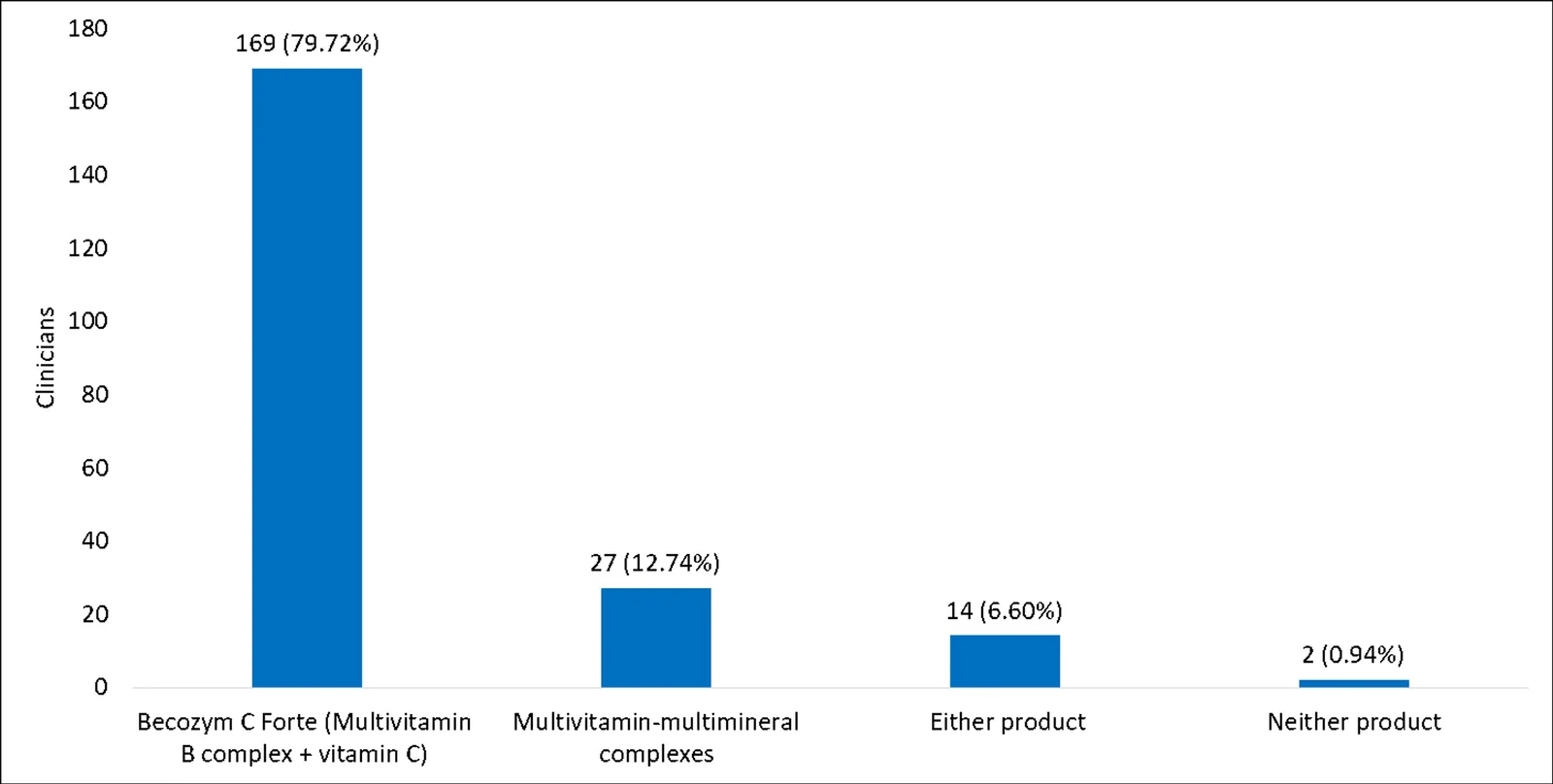
Prescribing preference for convalescing patients during acute illness or infection

In patients recovering from acute illness or infection, 79.72% (n=169) of clinicians preferred targeted B-complex plus vitamin C supplementation, while 12.74% (n=27) preferred multivitamin-multimineral products and 6.60% (n=14) reported no preference (Figure 6).

Peripheral Neuropathy or Nerve-Related Symptoms

**Figure 7:**
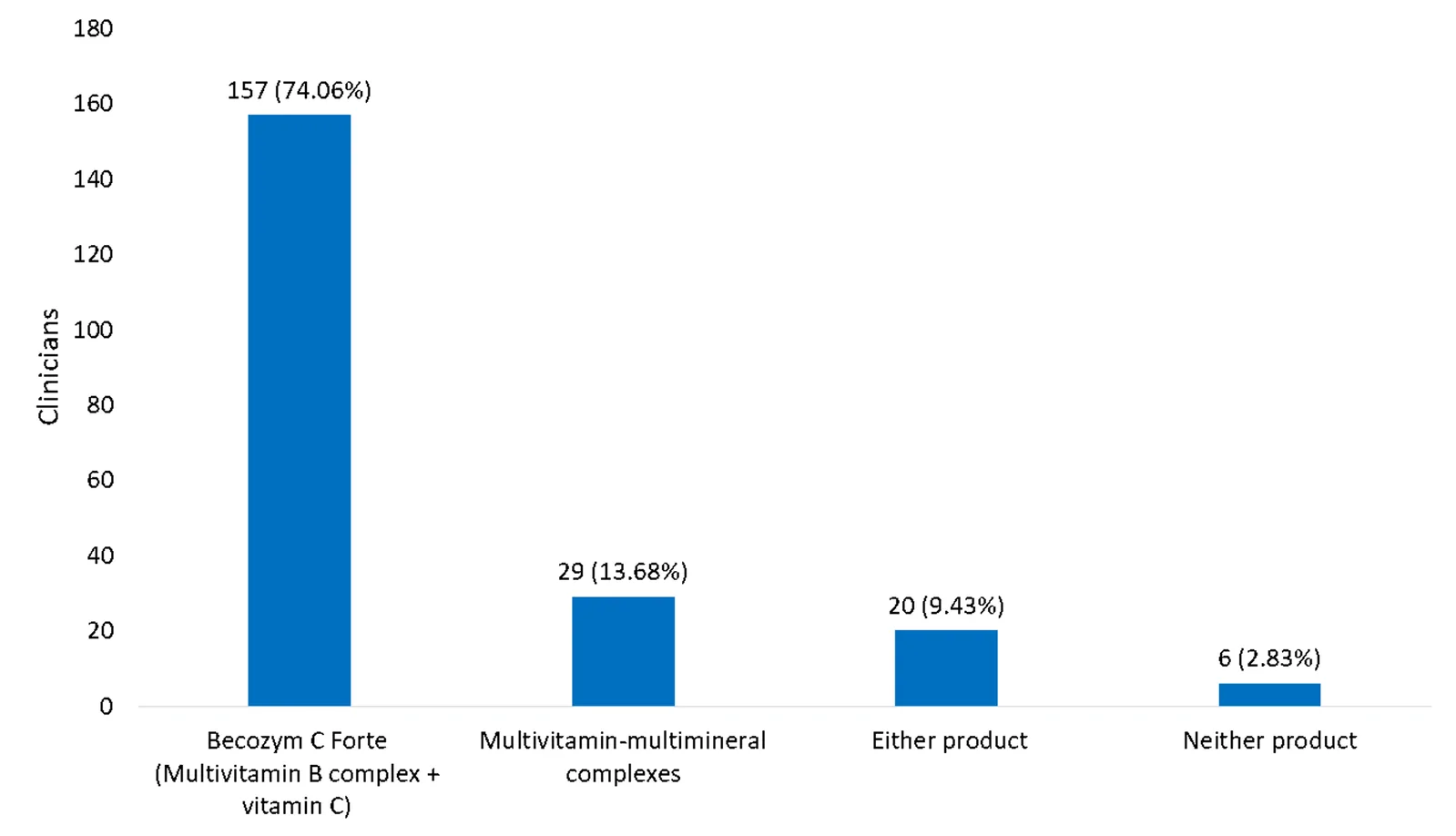
Prescribing preference for patients with peripheral neuropathy or nerve-related symptoms

For patients with neuropathy or nerve-related symptoms, 74.06% (n=157) of clinicians preferred targeted formulations, compared with 13.68% (n=29) favoring multivitamin-multimineral products and 9.43% (n=20) reporting no preference (Figure 7).

Recurrent Aphthous Ulcers

**Figure 8:**
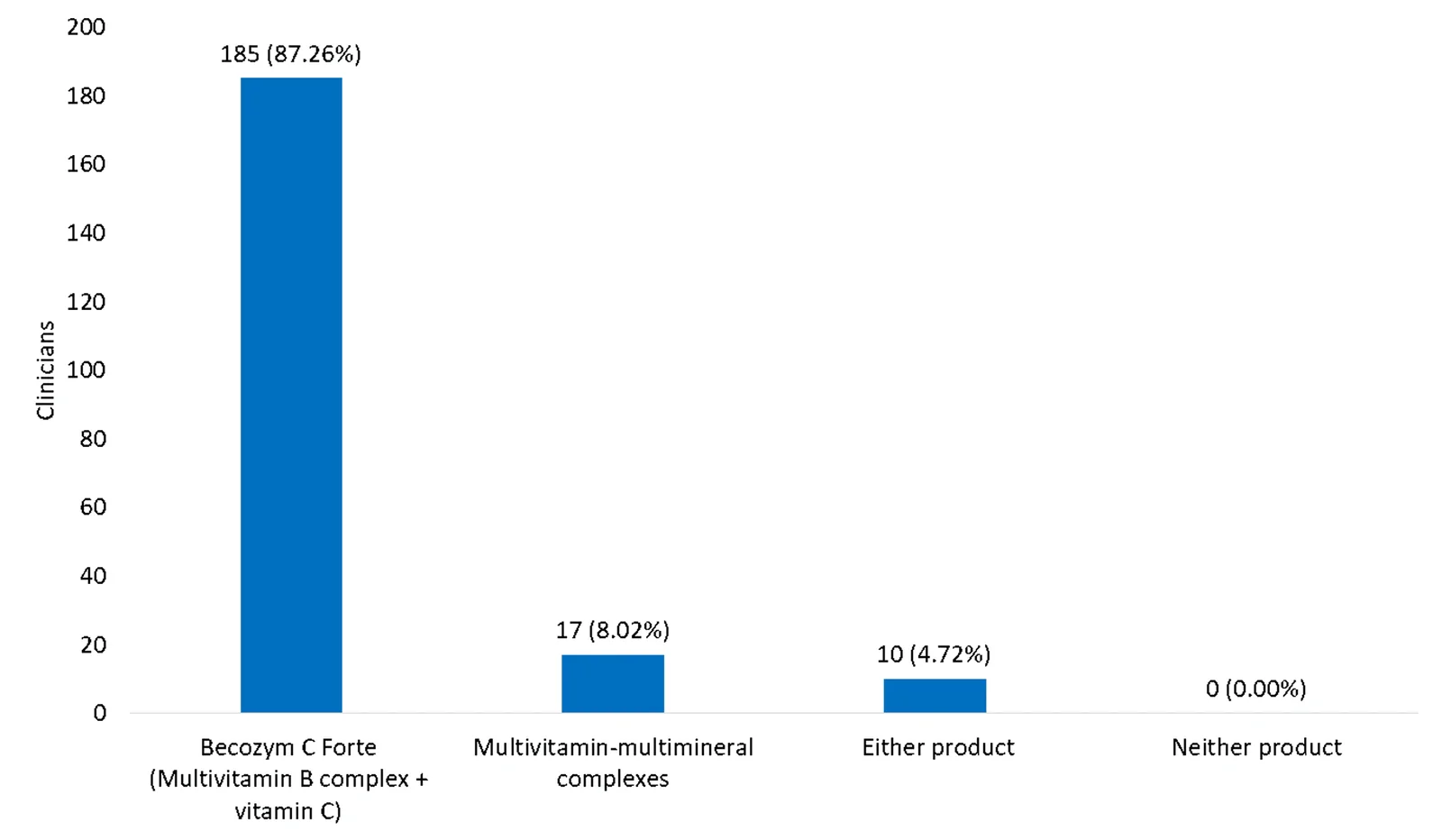
Prescribing preference for patients with recurrent aphthous ulcers

The strongest prescribing preference was observed in recurrent aphthous ulcers, where 87.26% (n=185) of clinicians selected targeted B-complex plus vitamin C formulations. Only 8.02% (n=17) preferred multivitamin-multimineral products, and 4.72% (n=10) reported no preference (Figure 8).

Malabsorption Syndromes

**Figure 9:**
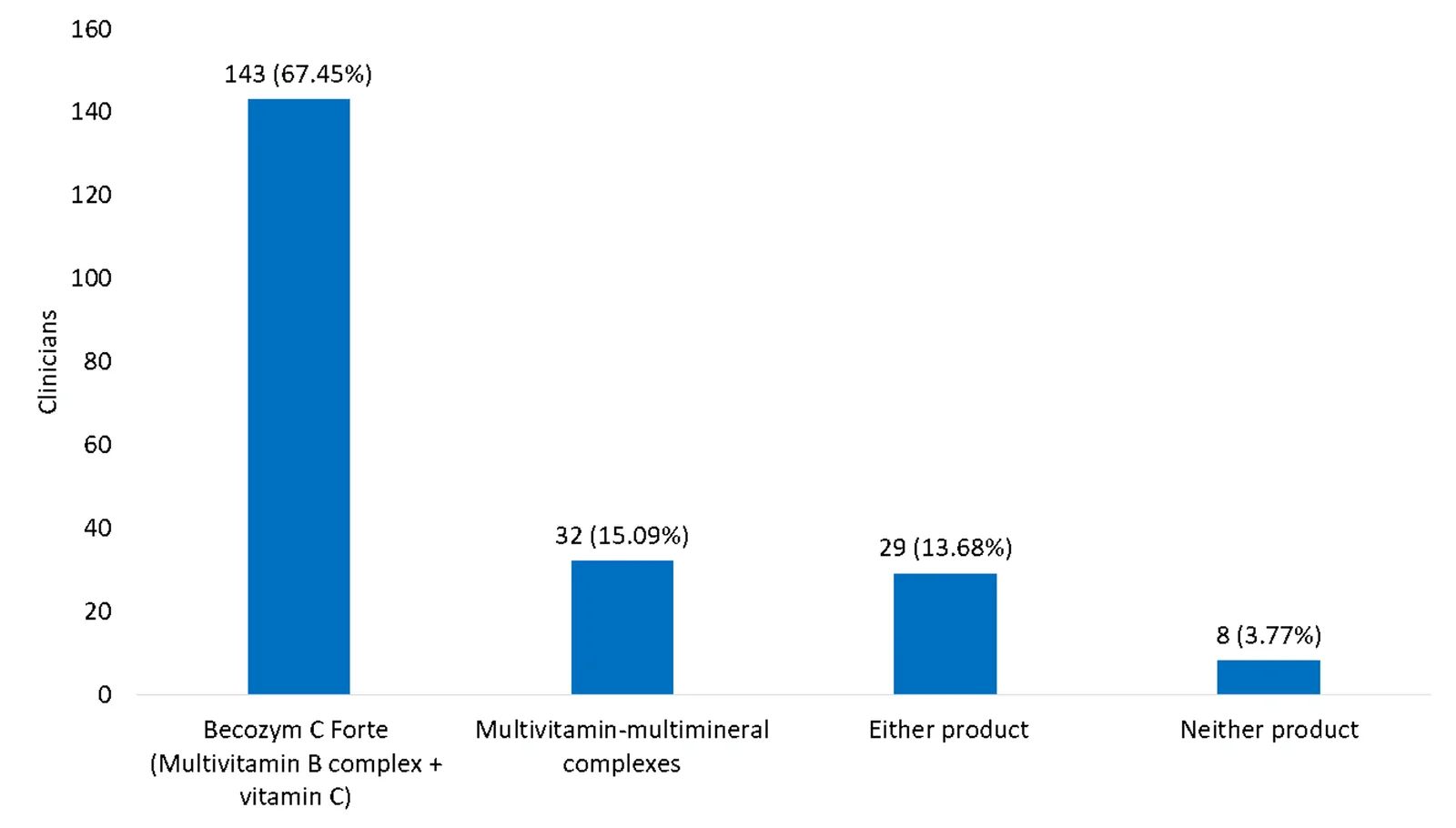
Prescribing preference for patients with malabsorption syndromes (IBD and celiac disease) IBD: Inflammatory Bowel Disease

In patients with malabsorption syndromes (e.g., inflammatory bowel disease and celiac disease), 67.45% (n=143) of clinicians preferred targeted formulations, while 15.09% (n=32) favored multivitamin-multimineral products and 13.68% (n=29) reported no preference (Figure 9).

Smokers and Chronic Alcohol Users

**Figure 10:**
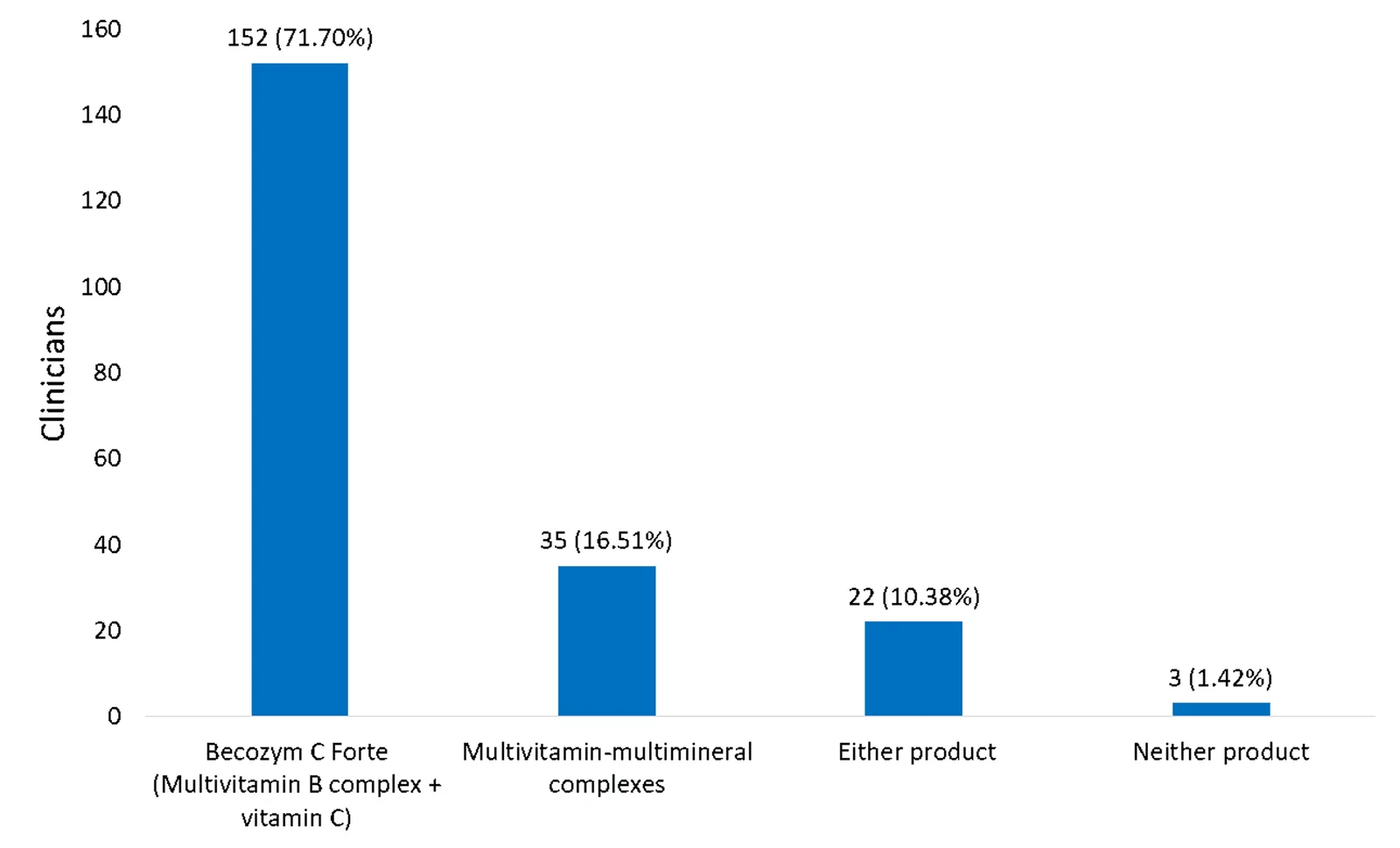
Prescribing preference for smokers and chronic alcohol users

Among populations with increased micronutrient depletion risk, such as smokers and chronic alcohol users, 71.70% (n=152) of clinicians preferred targeted B-complex plus vitamin C supplementation. Multivitamin-multimineral products were preferred by 16.51% (n=35), while 10.38% (n=22) reported no preference (Figure 10).

Diabetes and Metabolic Disorders

**Figure 11:**
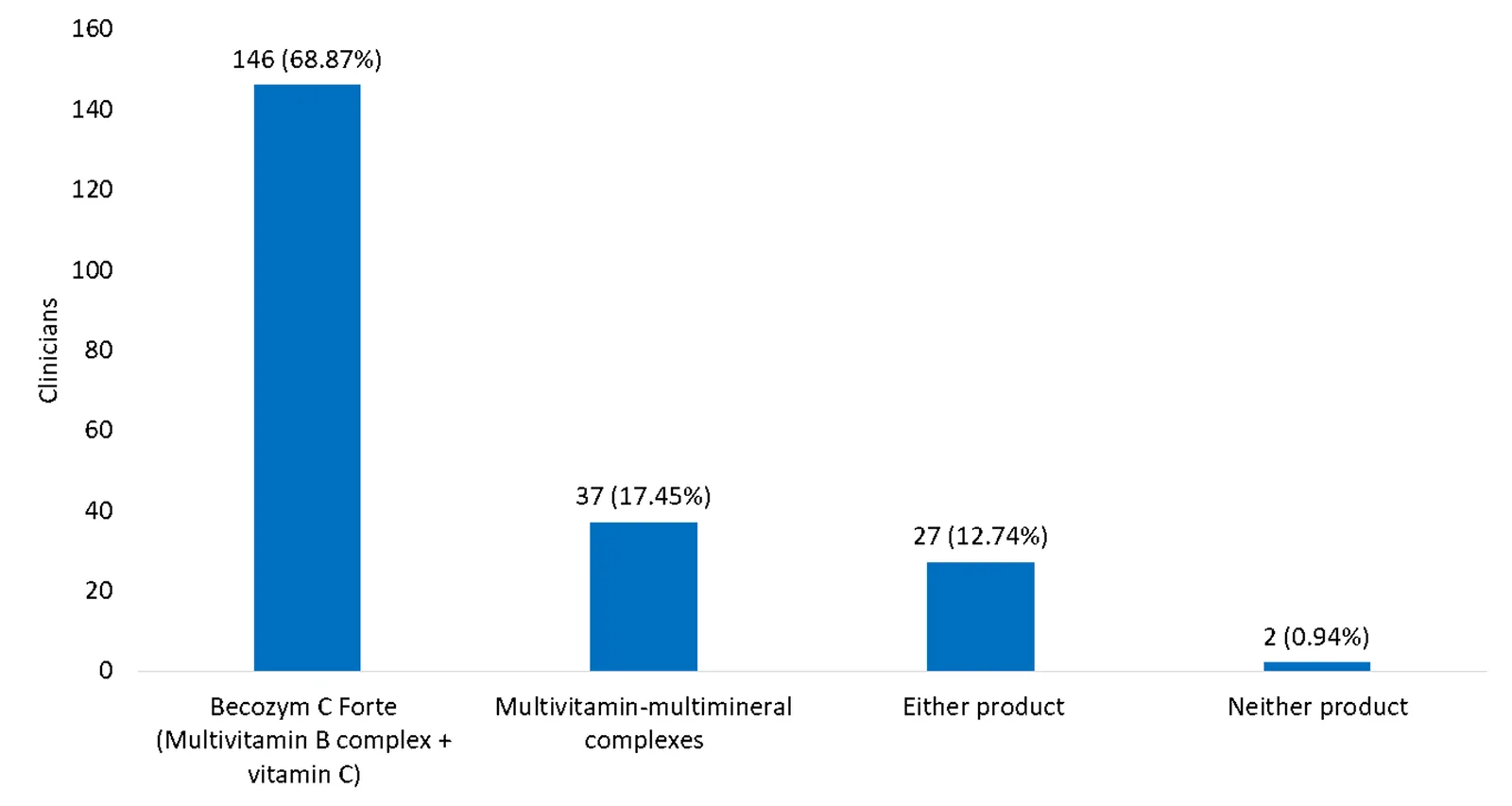
Prescribing preference for patients with diabetes or metabolic disorders

For patients with diabetes or metabolic disorders, 68.87% (n=146) of clinicians preferred targeted formulations, compared with 17.45% (n=37) for multivitamin-multimineral products and 12.74% (n=27) reporting no preference (Figure 11).

Acute Febrile Illness

**Figure 12:**
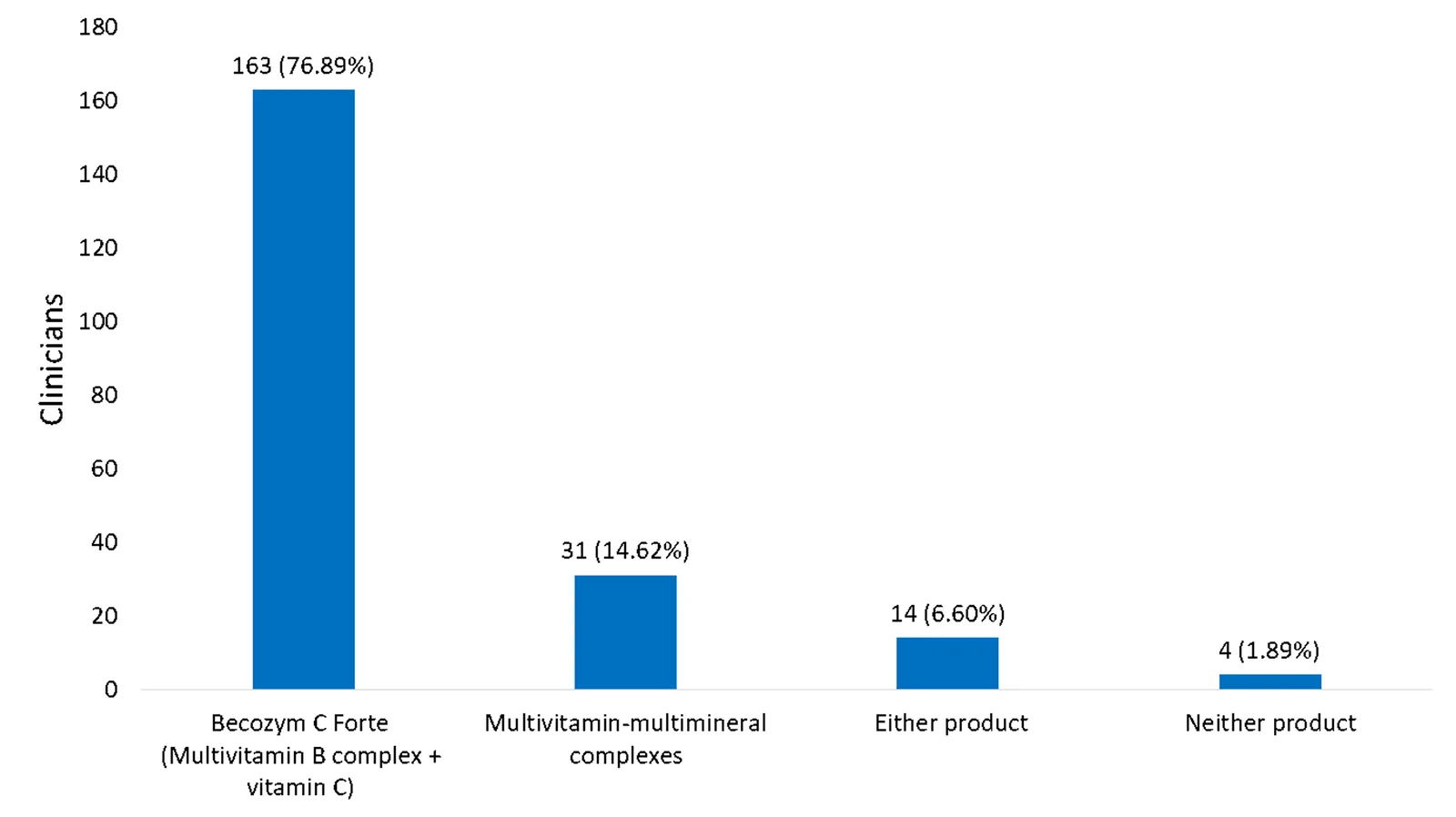
Prescribing preference for patients presenting with fever and malaise

In patients presenting with fever and malaise, 76.89% (n=163) of clinicians preferred targeted B-complex plus vitamin C supplementation, while 14.62% (n=31) preferred multivitamin-multimineral products and 6.60% (n=14) reported no preference (Figure 12).

Patients on Antibiotic Therapy

**Figure 13:**
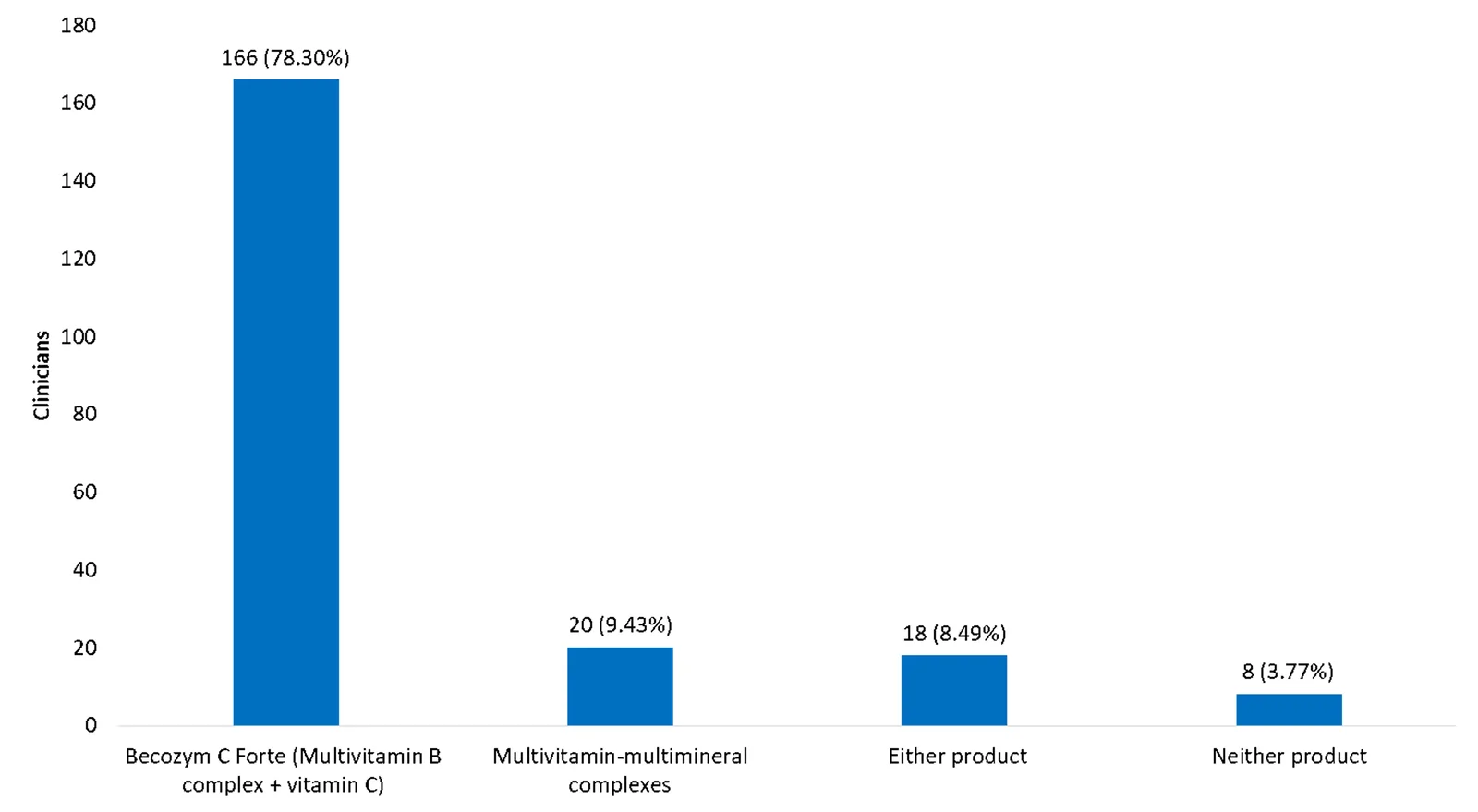
Prescribing preference for patients on a course of antibiotics

For patients receiving antibiotics, 78.30% (n=166) of clinicians preferred targeted formulations, compared with 9.43% (n=20) favoring multivitamin-multimineral products and 8.49% (n=18) reporting no preference (Figure 13).

Chronic Fatigue Syndrome or Fibromyalgia

**Figure 14:**
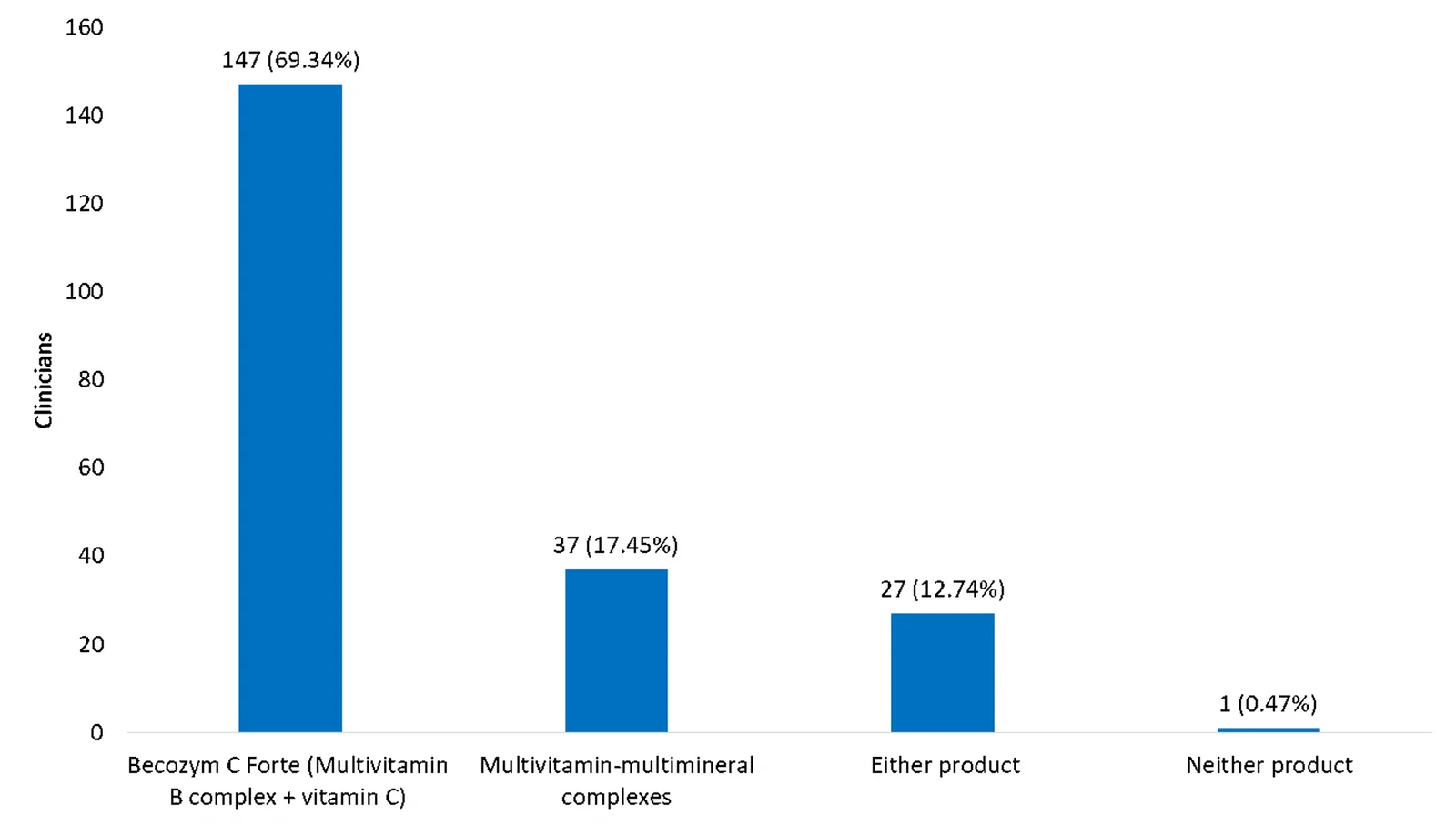
Prescribing preference for patients with CFS or fibromyalgia CFS: Chronic Fatigue Syndrome

In patients with chronic fatigue syndrome or fibromyalgia, 69.34% (n=147) of clinicians preferred targeted B-complex plus vitamin C supplementation, while 17.45% (n=37) preferred multivitamin-multimineral products and 12.74% (n=27) reported no preference (Figure 14).

Physician perceptions and comparative assessment

Observed Clinical Benefits Compared With Multivitamin-Multimineral Formulations

**Figure 15:**
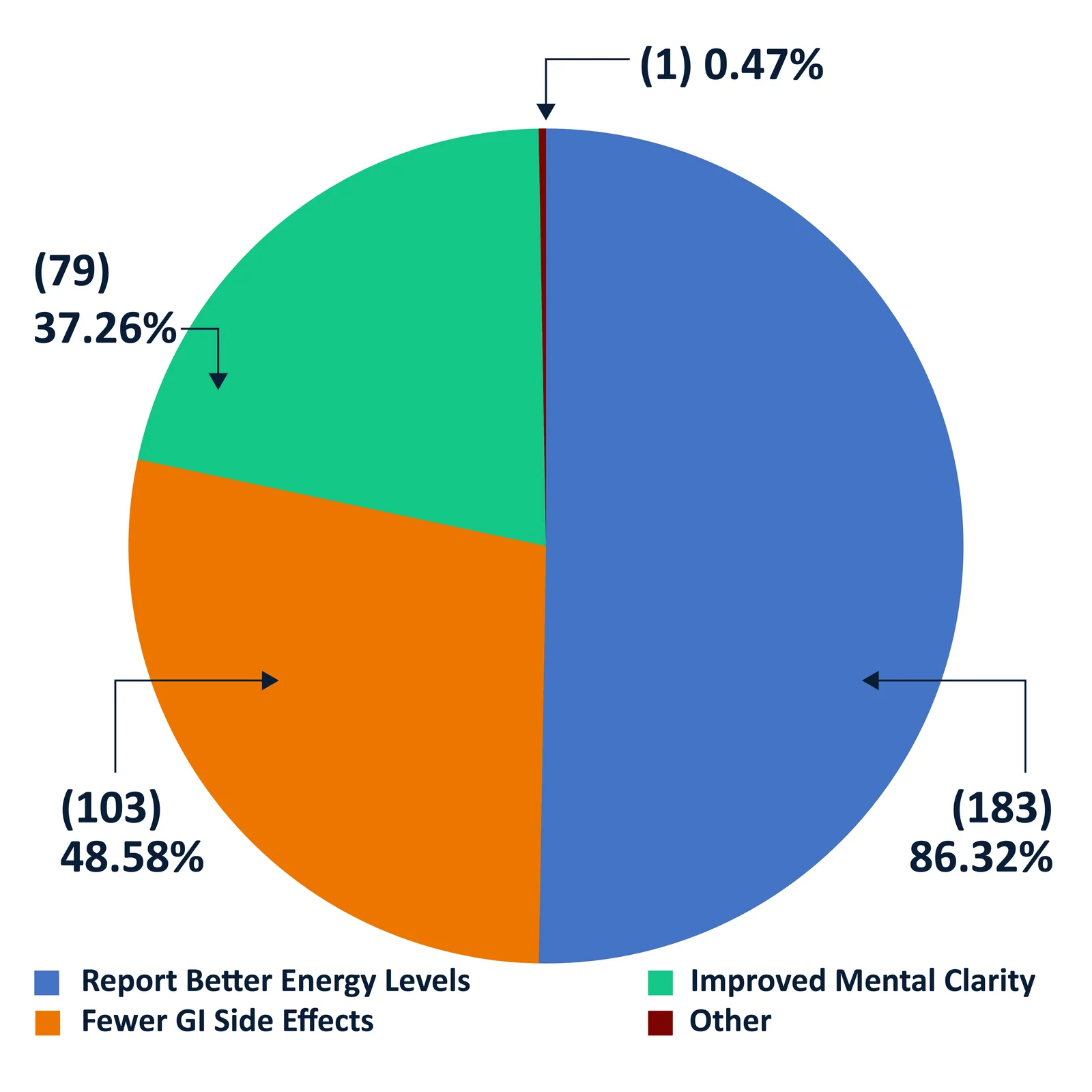
Clinician-observed patient benefits with focused B-complex + Vitamin C vs. multivitamin-multimineral formulations Multiple responses, n=212

Clinicians reported several perceived clinical advantages of targeted B-complex plus vitamin C supplementation compared with multivitamin-multimineral products. The most frequently reported benefit was improved energy levels, cited by 86.32% (n=183) of respondents. This was followed by fewer gastrointestinal side effects (48.58%, n=103) and improved mental clarity (37.26%, n=79). Only a negligible proportion (0.47%, n=1) reported other benefits. These findings suggest that clinicians commonly associate targeted formulations with enhanced tolerability and functional outcomes, particularly in domains related to energy and cognitive performance (Figure 15).

Perceived Advantages of Targeted Formulations

**Figure 16:**
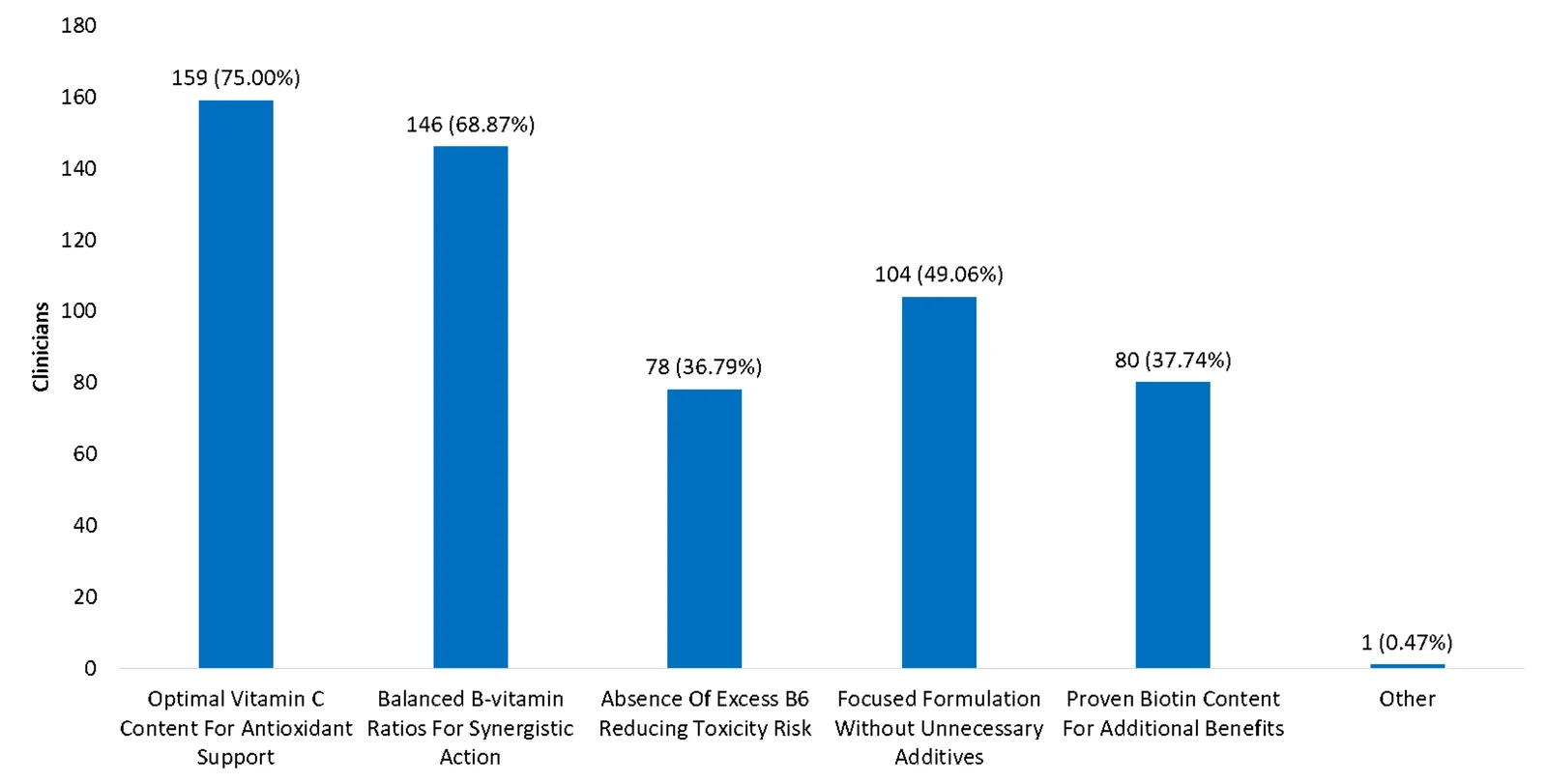
Factors influencing physician preference Multiple responses, n=212

When asked about factors contributing to the perceived clinical superiority of targeted B-complex plus vitamin C formulations, clinicians most frequently cited optimal vitamin C content for antioxidant support (75.00%, n=159). Balanced B-vitamin ratios for synergistic action were reported by 68.87% (n=146), while 49.06% (n=104) highlighted the advantage of a focused formulation without unnecessary additives. Additional attributes included proven biotin content (37.74%, n=80) and the absence of excess vitamin B6, reducing potential toxicity risk (36.79%, n=78). These responses indicate that both compositional specificity and safety considerations influence clinician preference for targeted formulations (Figure 16).

Perceptions on dosage form: tablet vs capsule

Tablet-Related Tolerability and Compliance

A large majority of clinicians (90.57%, n=192) agreed that tablet formulations are associated with less belching and improved patient compliance compared with capsules, while 9.43% (n=20) did not perceive a difference. This finding reflects a strong physician-perceived advantage of tablet formulations in terms of gastrointestinal tolerability and adherence. 

Overall Dosage Form Preference

**Figure 17:**
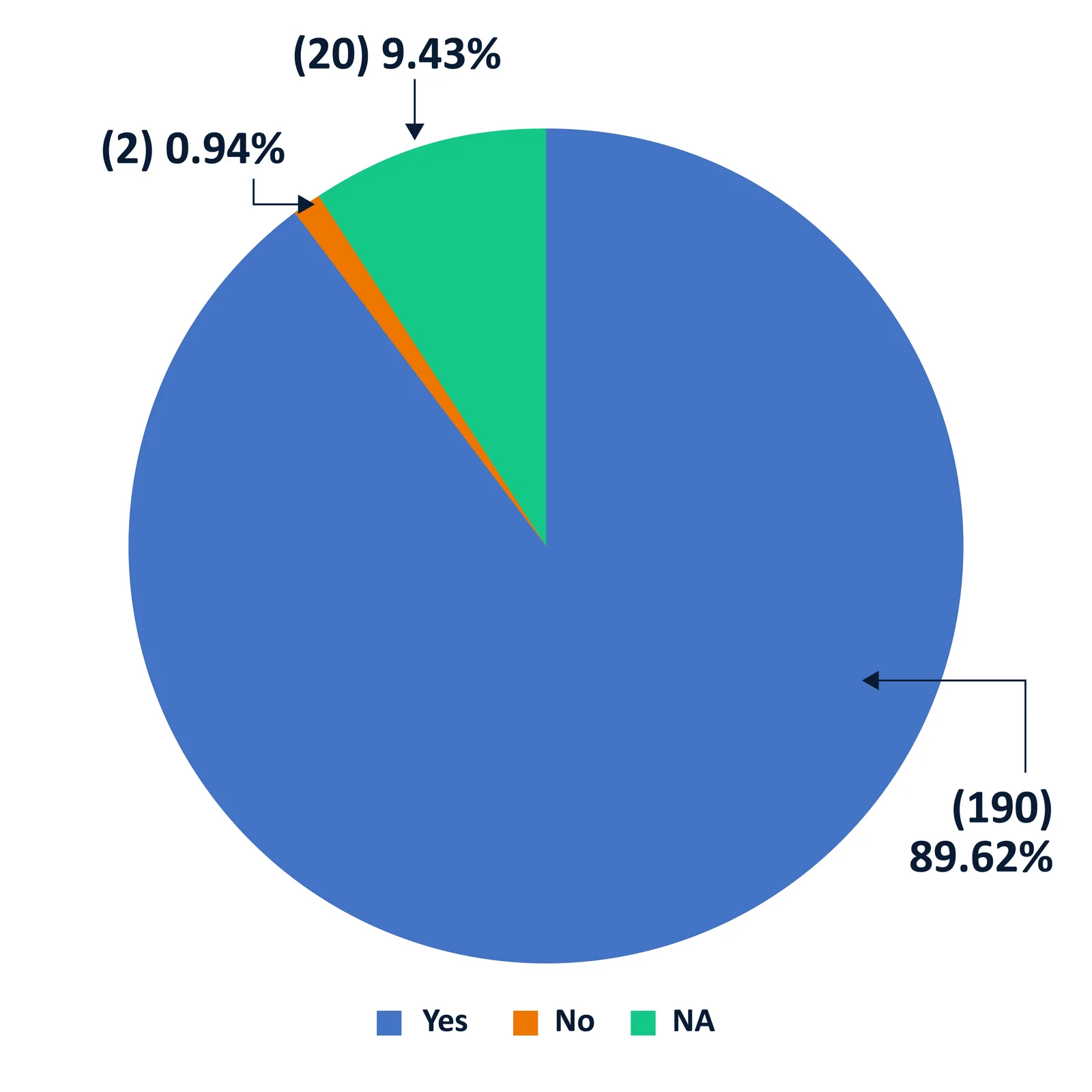
Clinician preference for tablet over capsule for multivitamin B-complex formulations

Consistent with the above observation, 89.62% (n=190) of clinicians reported a preference for tablet formulations over capsules for multivitamin B-complex supplementation. Only 0.94% (n=2) preferred capsules, while 9.43% (n=20) expressed no specific preference. This near-consensus preference further underscores the perceived practical advantages of tablet-based formulations in routine clinical use (Figure 17).

Overall, physicians demonstrated strong consensus favoring targeted B-complex + vitamin C formulations across clinical efficacy, patient outcomes, and prescribing scenarios. A single formulation emerged as the preferred choice, driven by its perceived advantages in energy support, tolerability, and formulation specificity. 

Heat map representation of physician responses

**Figure 18:**
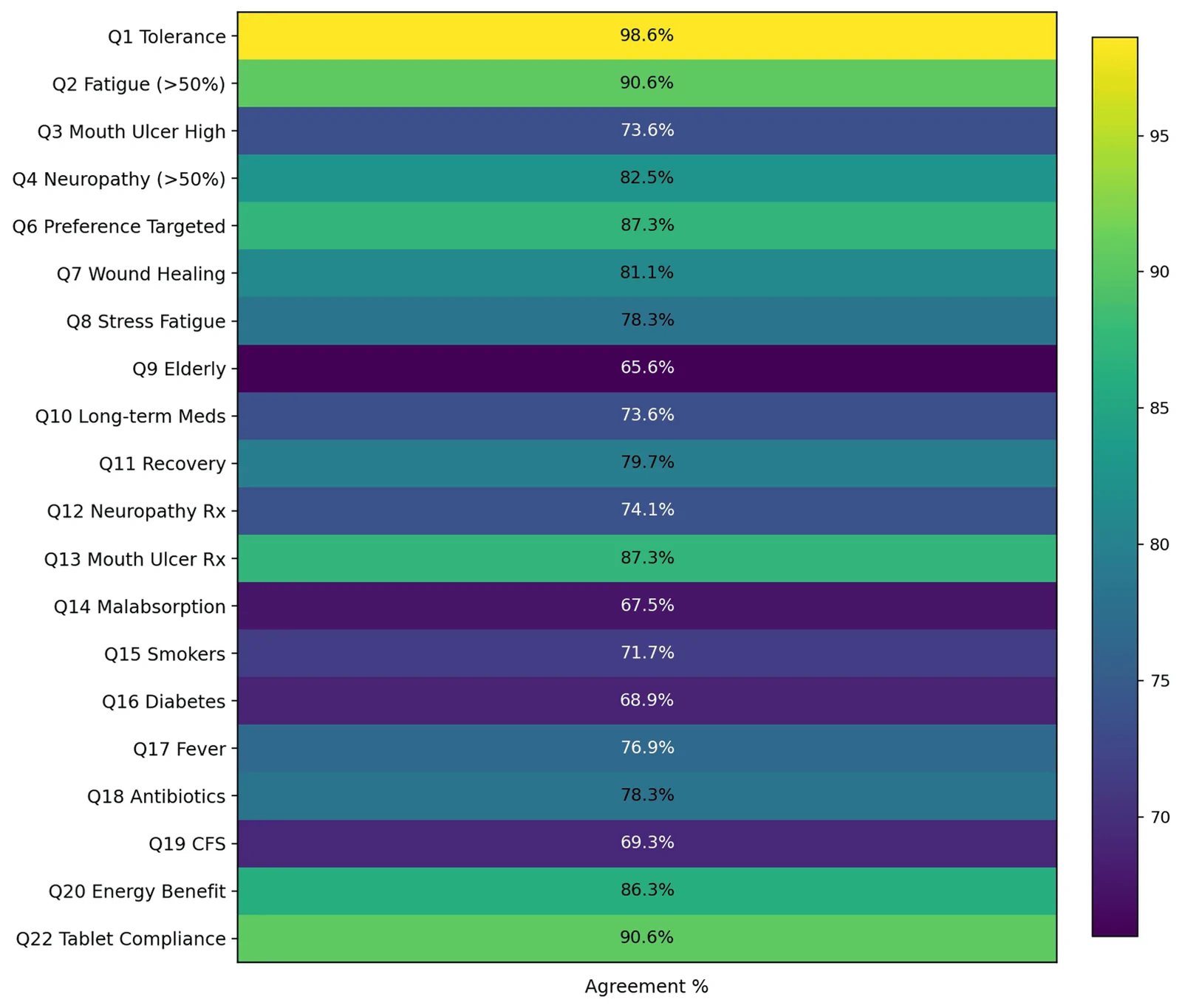
Heat map representation of physician responses across knowledge, attitudes, and practices (KAP) survey domains

This heat map illustrates the proportion (%) of clinicians reporting agreement, perceived efficacy, or prescribing preference for targeted B-complex + vitamin C formulations across individual survey questions (Q1-Q22; n=212). Color intensity corresponds to the magnitude of response, with higher values indicating stronger clinical consensus. The visualization highlights areas of high agreement (e.g., tolerance/adherence, fatigue relief, and tablet compliance) and comparatively lower consensus in specific clinical scenarios (e.g., elderly nutrition, malabsorption, and metabolic disorders), enabling rapid comparison across domains of clinical efficacy, prescribing preference, and physician perception (Figure 18).

## Discussion

The present KAP survey provides comprehensive real-world insights into physician perceptions and prescribing behaviors related to targeted B-complex and vitamin C supplementation. The findings demonstrate a high level of clinical consensus favoring targeted formulations across multiple domains, including tolerance, fatigue, oral health, neuropathy, and recovery-related conditions. These observations are largely consistent with established biological mechanisms and selected clinical evidence, while also reflecting prevailing prescribing trends in routine clinical practice.

The strong preference for targeted B-complex supplementation observed in this study is supported by the fundamental biological roles of B vitamins as coenzymes in a wide range of metabolic pathways, including energy production, DNA and RNA synthesis, methylation processes, and neurotransmitter regulation. Importantly, these vitamins function in a closely interdependent manner, and optimal physiological functioning requires adequate levels of the entire B-complex rather than isolated components. This interconnected role provides a mechanistic basis for the use of combined B-complex formulations, as reflected in the high clinician preference observed in the present survey [1,2].

Vitamin C further complements these effects through its potent antioxidant properties and its role as a cofactor in several biosynthetic and regulatory enzymatic processes. It contributes to immune defense by enhancing both innate and adaptive immune responses, supports epithelial barrier integrity, and is involved in collagen and carnitine synthesis, thereby linking immune function with tissue repair and energy metabolism. The combined use of B-complex and vitamin C therefore represents a biologically coherent approach targeting multiple interconnected physiological pathways [3].

The high proportion of clinicians reporting improvement in fatigue is consistent with the established role of B vitamins in intracellular energy metabolism. Although B vitamins do not directly provide energy, they act as essential cofactors in enzymatic pathways involved in ATP generation. Clinical evidence supports this mechanistic role, with randomized controlled data demonstrating improved exercise endurance and reduced fatigue-related biochemical markers following B-complex supplementation. These findings align with the observed clinician-reported benefits in fatigue and energy-related outcomes in the present study [4].

Similarly, the strong clinician-reported effectiveness in the management of mouth ulcers is supported by clinical evidence demonstrating the role of vitamin B12 in reducing pain and improving outcomes in aphthous ulcers. Randomized controlled data have shown significant reductions in pain scores with vitamin B12 therapy compared with placebo, supporting its role as an adjunctive treatment in oral mucosal conditions. This is consistent with the high level of preference for targeted B-complex formulations in this indication observed in the survey [7].

In the context of neuropathy and nerve-related symptoms, clinicians in the present study reported perceived benefits with B-complex supplementation. This observation is biologically plausible, as vitamin B12 deficiency is strongly associated with peripheral neuropathy and elevated homocysteine levels. However, evidence from interventional studies remains heterogeneous. Systematic reviews and meta-analyses suggest that B-vitamin deficiency is associated with neuropathy, and supplementation may offer clinical benefits in selected patient populations. Accordingly, the observations from this study likely reflect real-world physician experiences and perceptions regarding effectiveness [5].

The preference for targeted supplementation in recovery-related conditions, including acute illness, antibiotic use, and wound healing, can be explained by the complementary roles of B vitamins and vitamin C in immune function and tissue repair. Vitamin C plays a central role in immune cell function, oxidative stress reduction, and collagen synthesis, while experimental evidence suggests that B-vitamin supplementation may enhance wound healing and tissue regeneration. These combined effects provide a plausible explanation for the observed clinician preference in such clinical scenarios [3,8].

The study also demonstrates consistent physician preference for targeted supplementation in populations at increased risk of micronutrient deficiency, including elderly individuals, patients with malabsorption, and those with chronic diseases such as diabetes. This aligns with evidence indicating that B-vitamin requirements vary across the life cycle and that deficiency risk increases in conditions of increased metabolic demand or impaired absorption. Additionally, long-term use of medications such as metformin has been associated with vitamin B12 deficiency, further supporting the rationale for supplementation in these patient groups [2]. Real-world physician surveys similarly highlight persisting gaps in B12 screening and supplementation practices among metformin-treated patients with diabetes [9]. Notably, vitamin B12 deficiency is estimated to affect 10%-15% of adults over 60 years of age, largely due to age-related declines in gastric acid secretion and intrinsic factor availability that impair absorption of protein-bound B12 [10]. A systematic review and meta-analysis confirmed a significantly higher prevalence of vitamin B12 deficiency among metformin-treated patients compared with non-users, reinforcing the clinical relevance of monitoring and supplementation in this population [11]. Smokers and chronic alcohol users represent a further at-risk group, as oxidative stress and altered nutrient turnover associated with smoking are consistently linked to lower circulating vitamin C concentrations even after adjustment for dietary intake [12]. In chronic fatigue syndrome and fibromyalgia, evidence for vitamin and mineral deficiencies as a unifying cause of symptoms remains inconsistent according to a systematic review and meta-analysis, though micronutrient support continues to be considered as part of broader fatigue management strategies, consistent with the preference observed in this survey [13].

A notable finding of this study is the strong preference for targeted B-complex and vitamin C formulations over multivitamin-multimineral products. This may reflect a clinical inclination toward more focused and physiologically relevant supplementation strategies. Evidence regarding multivitamin supplementation remains inconsistent, particularly in relation to functional and cognitive outcomes, whereas targeted approaches may better address specific deficiencies and metabolic needs.

These findings should also be interpreted in the context of broader prescribing practices. Vitamins constitute a significant proportion of prescriptions in clinical practice, accounting for approximately one-fourth of all prescribed drugs in India, often in the absence of clear evidence-based indications. This highlights a gap between widespread clinical use and the strength of supporting evidence. While biological plausibility and selective clinical studies support the use of B-complex and vitamin C in certain conditions, evidence remains heterogeneous for several indications, particularly neuropathy and chronic disease outcomes. The present study therefore underscores the need for more robust, indication-specific clinical trials to better align clinical practice with evidence-based recommendations [4].

Tuberculosis (TB) continues to be a major public health challenge in India and is frequently associated with malnutrition, weight loss, fatigue, anorexia, and micronutrient deficiencies. Furthermore, patients receiving anti-TB therapy may experience treatment-related adverse effects such as peripheral neuropathy, particularly with isoniazid-containing regimens. B-complex vitamin supplementation is routinely used in clinical practice to support nutritional recovery and reduce the risk of neuropathy, while vitamin C has been explored for its antioxidant and immunomodulatory properties and has demonstrated antimycobacterial activity in experimental models, although clinical relevance remains to be established. The present survey did not specifically assess patients with TB; the strong physician preference for targeted B-complex and vitamin C supplementation in conditions characterized by fatigue, nutritional compromise, and neuropathic symptoms suggests potential relevance in the supportive care of patients receiving anti-TB treatment. Further prospective studies are needed to evaluate these benefits in TB-specific populations [14,15].

In summary, the findings of this KAP survey reflect a strong physician preference for targeted B-complex and vitamin C supplementation across a range of clinical scenarios. These perceptions are supported by established biological mechanisms and selective clinical evidence, while also highlighting areas where further research is needed to bridge the gap between real-world practice and high-quality evidence.

Limitations

Several limitations of this study should be considered when interpreting the findings. First, the survey evaluated physician perceptions, prescribing preferences, and self-reported clinical experiences rather than patient-level clinical outcomes. Consequently, the reported benefits reflect clinician observations and may not directly correspond to objectively measured treatment effects.

Second, the cross-sectional survey design provides a snapshot of current prescribing practices and perceptions but does not allow assessment of causality or longitudinal outcomes. Third, responses may be subject to recall bias, as clinicians were asked to report experiences from routine practice, and selection bias may have occurred because participation was voluntary and limited to physicians who chose to respond to the survey. Additionally, the questionnaire was not formally pretested or validated prior to deployment, which may have affected item clarity or interpretation.

In addition, the study did not include objective measures of clinical efficacy, laboratory parameters, or patient-reported outcomes. Therefore, comparisons between focused micronutrient formulation and multivitamin-multimineral formulations should be interpreted cautiously. The findings may also have limited generalizability beyond the surveyed physician population and may not fully represent prescribing practices across all healthcare settings or specialties in India.

Furthermore, the survey was industry-sponsored, which may introduce the potential for sponsorship-related bias despite the use of standardized survey methods and anonymous data collection.

Finally, although the survey provides valuable real-world insights into physician perceptions and prescribing preferences, prospective clinical studies and randomized controlled trials are needed to validate the perceived benefits and establish the clinical effectiveness of targeted B-complex and vitamin C supplementation across different patient populations.

## Conclusions

Physicians reported a strong preference for targeted B-complex and vitamin C supplementation across multiple clinical scenarios, including fatigue, oral health conditions, recovery-related states, and suspected water-soluble vitamin deficiencies. These findings reflect real-world prescribing perceptions and experiences rather than objective measures of clinical effectiveness. While the reported benefits are broadly consistent with the known biological roles of B-complex vitamins and vitamin C, the results should be interpreted in conjunction with the available clinical evidence and the inherent limitations of survey-based research. Further prospective studies are warranted to better define the clinical effectiveness and optimal use of targeted supplementation in routine practice.

## Appendices

Appendix 1

**Table 7:** Survey questionnaire

No.	Questions	Answer options
A	Clinical Efficacy and Patient Outcomes	
1	In your view, could a focused B-complex and Vitamin C formulation, which minimizes inter-nutrient interactions relative to multivitamin-multimineral products, translate into better patient tolerance and adherence?	A) Yes, B) No
2	Based on your clinical experience, what proportion of your patients report relief from fatigue within 2-3 weeks of B-Complex therapy?	A) 75% of patients B) 50-75% of patients C) 25-50% of patients D) <25% of patients
3	Considering that B-complex supplementation helps reduce the frequency and duration of mouth ulcers, how would you rate its effectiveness in achieving relief within 7 days?	A) Highly effective (>75% of patients respond) B) Moderately effective (50-75% respond) C) Somewhat effective (25-50% respond) D) Not effective (<25% respond)
4	What proportion of your patients with neuropathy/nerve-related symptoms report improvement with B-Complex supplementation?	A) 75% B) 50-75% C) 25-50% D) <25%
B	Product Preference for Clinical Scenarios	
5	For which conditions do you most commonly recommend B-Complex + vitamin C without added multiminerals? Tick multiple options which apply	A) Nerve health, B) Mouth ulcers, C) General Fatigue, D) Acute illness for faster recovery, E) Immune support, F) Wound healing support, G) Stress management, H) Anaemia, I) Cognitive Health
6	When prescribing for suspected "specific" B-vitamin and vitamin C deficiencies (water-soluble vitamins), what do you prefer?	A) Focused B-complex + Vitamin C formulations, B) Comprehensive multivitamin-multimineral complexes
7	For the following patient scenario, which product would you preferentially prescribe? Post-surgical/ post-injury recovery patient needing wound healing support	A) Becozym C Forte (Multivitamin B complex + vitamin C), B) Multivitamin-multimineral complexes, C) Either product, D) Neither product
8	For the following patient scenario, which product would you preferentially prescribe? Stressed professional with fatigue and concentration issues	A) Becozym C Forte (Multivitamin B complex + vitamin C), B) Multivitamin-multimineral complexes, C) Either product, D) Neither product
9	For the following patient scenario, which product would you preferentially prescribe? Elderly patient with poor dietary intake	A) Becozym C Forte (Multivitamin B complex + vitamin C), B) Multivitamin-multimineral complexes, C) Either product, D) Neither product
10	For the following patient scenario, which product would you preferentially prescribe? Patients on Long-Term Medications (e.g., Metformin, PPIs, Anticonvulsants)	A) Becozym C Forte (Multivitamin B complex + vitamin C), B) Multivitamin-multimineral complexes, C) Either product, D) Neither product
11	For the following patient scenario, which product would you preferentially prescribe? Convalescing Patients During Acute Illness or Infection	A) Becozym C Forte (Multivitamin B complex + vitamin C), B) Multivitamin-multimineral complexes, C) Either product, D) Neither product
12	For the following patient scenario, which product would you preferentially prescribe? Patients with Peripheral Neuropathy or Nerve-Related Symptoms	A) Becozym C Forte (Multivitamin B complex + vitamin C), B) Multivitamin-multimineral complexes, C) Either product, D) Neither product
13	For the following patient scenario, which product would you preferentially prescribe? Patients with Recurrent Aphthous Ulcers (Mouth Ulcers)	A) Becozym C Forte (Multivitamin B complex + vitamin C), B) Multivitamin-multimineral complexes, C) Either product, D) Neither product
14	For the following patient scenario, which product would you preferentially prescribe? Patients with Malabsorption Syndromes (e.g., IBD, Celiac Disease)	A) Becozym C Forte (Multivitamin B complex + vitamin C), B) Multivitamin-multimineral complexes, C) Either product, D) Neither product
15	For the following patient scenario, which product would you preferentially prescribe? Smokers and Chronic Alcohol Users	A) Becozym C Forte (Multivitamin B complex + vitamin C), B) Multivitamin-multimineral complexes, C) Either product, D) Neither product
16	For the following patient scenario, which product would you preferentially prescribe? Patients with Diabetes or Metabolic disorders (obesity, dyslpidemia, Fatty Liver Disease)	A) Becozym C Forte (Multivitamin B complex + vitamin C), B) Multivitamin-multimineral complexes, C) Either product, D) Neither product
17	For the following patient scenario, which product would you preferentially prescribe? Patients with fever, malaise	A) Becozym C Forte (Multivitamin B complex + vitamin C), B) Multivitamin-multimineral complexes, C) Either product, D) Neither product
18	For the following patient scenario, which product would you preferentially prescribe? Patients on a course of antibiotic	A) Becozym C Forte (Multivitamin B complex + vitamin C), B) Multivitamin-multimineral complexes, C) Either product, D) Neither product
19	For the following patient scenario, which product would you preferentially prescribe? Patients with Chronic Fatigue Syndrome (CFS) or Fibromyalgia	A) Becozym C Forte (Multivitamin B complex + vitamin C), B) Multivitamin-multimineral complexes, C) Either product, D) Neither product
C	Physician Perceptions & Comparative Assessment	
20	Based on your observation, patients using focused B-complex + vitamin C supplementation (such as Becozym C Forte), compared to multimineral–multivitamin formulations, show which of the following (Choose all that applies)	A)☐ Report better energy levles B)☐ fewer GI side effects C)☐ Improved mental calrity D)☐ Other______________
21	What makes Becozym C Forte potentially (clinically) superior to competitors? (Select all applicable):	A) ☐ Optimal vitamin C content for antioxidant support B) ☐ Balanced B-vitamin ratios for synergistic action C) ☐ Absence of excess B6 reducing toxicity risk D) ☐ Focused formulation without unnecessary additives E) ☐ Proven biotin content for additional benefits F) ☐ Other____________________________
22	Do you think that tablet forms cause less belching and have better compliance compared to capsules?	A) Yes, B) No
	If yes is selected, the below question will pop-up.	
23	Would you prefer a tablet for multivitamin B complex over a capsule?	A) Yes, B) No
